# Foreign Correspondence

**Published:** 1889-07

**Authors:** H. H. Edwards

**Affiliations:** Madrid, Spain


### Foreign Correspondence.

To the Editor:

Incidents of practice are ever-welcome reading, because every now and then occurs a case which, to the best of us, presents a difficulty *pro tempore*, and is only to be conquered by a happy inspiration not to be met with in text-books.

Such a case presented itself to me the other day. A patient came complaining of hypersensibility of the second right upper molar. It stood alone, occluding with its fellow below, was slightly loose, had no decay, but so sensitive to heat and cold—on account of the roots being denuded—that eating and drinking was simply a misery. I tried all the means I knew to reduce the sensibility, but without avail. Killing the pulp or extracting the molar were means I hesitated to employ except as a last resource.

The suggestion of wearing artificial teeth did not meet with his approval, by which means the tooth would have been covered with the plate, and would have no doubt improved the situation. I took, as an experiment, cotton-wool and mastic, and tied a strand around the molar; the result being satisfactory, I took an impression of the molar with wax—being preferable to modelling compound on account of the temperature necessary—and made a very thin shell of vulcanite perfectly fitting the tooth to the margin of the gums, leaving the crown exposed for the purpose of articulation.

The shell or overcoat, cemented on with oxyphosphate, has been in wear a month, and has completely frustrated contact with heat or cold. I may say it has proved a success. By such simple means I have been enabled to save the molar alive and receive the thanks and confidence of my patient.

H. H. Edwards.

Madrid, Spain, May 20, 1889.

